# Prevalence of molecular markers of sulfadoxine–pyrimethamine and artemisinin resistance in *Plasmodium falciparum* from Pakistan

**DOI:** 10.1186/s12936-018-2620-y

**Published:** 2018-12-17

**Authors:** Adnan Yaqoob, Aamer Ali Khattak, Muhammad Faisal Nadeem, Huma Fatima, Gillian Mbambo, Amed Ouattara, Matthew Adams, Nadia Zeeshan, Shannon Takala-Harrison

**Affiliations:** 1 Department of Biochemistry & Biotechnology, University of Gujrat, Gujrat, Pakistan; 2 Department of Medical Laboratory Technology, University of Haripur, Haripur, KPK Pakistan; 3 Department of Animal Sciences, Quaid-i-Azam University, Islamabad, Pakistan; 4 Center for Vaccine Development and Global Health, University of Maryland School of Medicine, Baltimore, USA

**Keywords:** Malaria, *Plasmodium falciparum*, Drug resistance, Pakistan

## Abstract

**Background:**

In Pakistan, artesunate (AS) in combination with sulfadoxine–pyrimethamine (SP) is the recommended treatment for uncomplicated *Plasmodium falciparum* malaria. Monitoring molecular markers of anti-malarial drug resistance is crucial for early detection and containment of parasite resistance to treatment. Currently, no data are available on molecular markers of artemisinin resistance (K13 mutations) in *P. falciparum* isolates from Pakistan. In this study, the prevalence of mutations associated with SP and artemisinin resistance was estimated in different regions of Pakistan.

**Methods:**

A total of 845 blood samples that were positive for malaria parasites by microscopy or rapid diagnostic test were collected from January 2016 to February 2017 from 16 different sites in Pakistan. Of these samples, 300 were positive for *P. falciparum* by PCR. Polymorphisms in the *P. falciparum* dihydrofolate reductase (*pfdhfr*) and dihydropteroate synthase (*pfdhps*) genes were identified by pyrosequencing while polymorphisms in the propeller domain of the *pfk13* gene were identified by Sanger sequencing.

**Results:**

The prevalence of the PfDHFR 108N and 59R mutations was 100% and 98.8%, respectively, while the prevalence of PfDHFR 50R and 51I mutations was 8.6%. No mutation was observed at PfDHFR position 164. In PfDHPS, the prevalence of mutations at positions 436, 437, and 613 was 9.9%, 45.2%, and 0.4%, respectively. No mutations were found at PfDHPS positions 540 and 581. The prevalence of double PfDHFR mutants (59R + 108N) ranged from 93.8% to 100%, while the prevalence of parasites having the PfDHFR 59R + 108N mutations in addition to the PfDHPS 437G mutation ranged from 9.5% to 83.3% across different regions of Pakistan. Nine non-synonymous and four synonymous mutations were observed in the PfK13 propeller domain, none of which correspond to mutations validated to contribute to artemisinin resistance.

**Conclusion:**

The absence of the highly resistant PfDHFR/PfDHPS quintuple mutant parasites and the lack of PfK13 mutations associated with artemisinin resistance is consistent with AS + SP being effective in Pakistan.

**Electronic supplementary material:**

The online version of this article (10.1186/s12936-018-2620-y) contains supplementary material, which is available to authorized users.

## Background

Malaria is a devastating disease affecting people in 91 countries with about half of the global population at risk [1]. In 2016, an estimated 216 million cases of malaria occurred worldwide resulting in an estimated 445,000 deaths [1]. Malaria transmission is moderate in Pakistan with 177 million people at risk [2]. *Plasmodium vivax* is the most prevalent species of *Plasmodium* in Pakistan, accounting for 79% of malaria cases in 2016, followed by *Plasmodium falciparum* which was responsible for the remaining 21% of malaria cases [1]. The emergence and spread of resistance to anti-malarial drugs is challenging for malaria control [3]. *Plasmodium falciparum* has developed resistance to all anti-malarial drugs during the past 50 years [4]. In Pakistan, artemisinin-based combination therapy (ACT) with artesunate plus sulfadoxine–pyrimethamine (AS + SP) has been the first-line treatment for uncomplicated falciparum malaria since 2007.

Studies have shown that single nucleotide polymorphisms (SNPs) in the dihydrofolate reductase (*pfdhfr*) and dihydropteroate synthase (*pfdhps*) genes of *P. falciparum* confer resistance to pyrimethamine and sulfadoxine, respectively [5–8]. PfDHFR mutations C50R, N51I, C59R, S108N, and I164L are associated with resistance to pyrimethamine, while PfDHPS mutations S436A/F, A437G, K540E, A581G and A613S/T are associated with resistance to sulfadoxine [9–13]. The presence of a triple mutation in PfDHFR (51I+59R+108N) has been associated with SP treatment failure [14–16], as has the combination of PfDHFR triple mutant (51I+59R+108N) and PfDHPS double mutant (437G+540E) (i.e. the “quintuple mutant”) [17, 18]. A high prevalence of PfDHFR double mutants (59R + 108N) ranging from 87 to 100% has been reported in previous studies in Pakistan [19–22]. This PfDHFR double mutant (59R + 108N) in combination with the PfDHPS 437G mutation is the next most prominent combination of mutations reported in Pakistan with prevalence ranging from 31.8 to 69% [19–22].

Artemisinin resistance has been detected along the Thailand-Cambodia border, in Vietnam, Myanmar, and other countries in Southeast Asia [23]. Delayed parasite clearance was first observed in *P. falciparum*-infected patients in Cambodia about 10 years ago [24, 25]. Specific mutations in the propeller domain of the PfK13 protein (PF3D7_1343700) have been shown to confer resistance to the artemisinins [26, 27], with several different polymorphisms associated with the resistant phenotype [23, 28, 29]. This study aims to investigate the prevalence of PfDHFR and PfDHPS alleles associated with SP resistance and to identify SNPs located within the PfK13 protein in Pakistan.

## Methods

### Study sites and ethical considerations

**Fig. 1:**
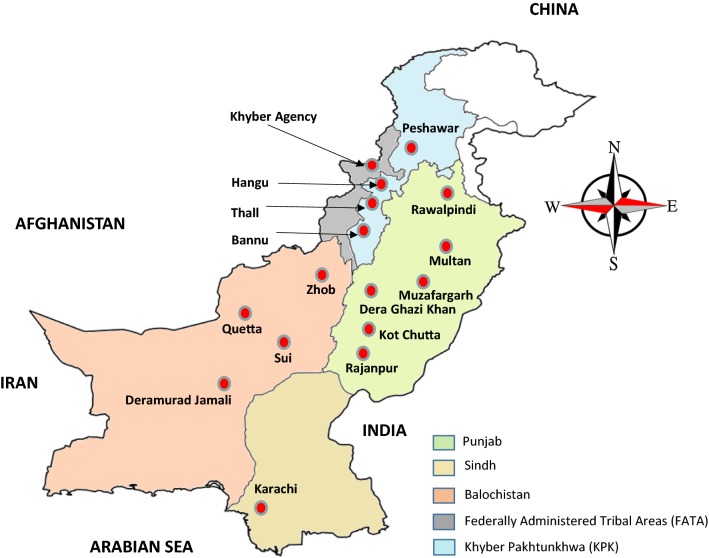
Geographic location of sample collection sites in 16 different areas of Pakistan. Red dots indicate the areas from where samples were collected

Symptomatic febrile patients who visited government or private hospitals at 16 different sites within Pakistan from January 2016 to February 2017 were enrolled in the study (Fig. 1). Informed consent and/or assent were obtained from study participants or their legal guardian. Demographic characteristics including age and gender were recorded. The study was approved by the Institutional Review Board of the University of Gujrat, Pakistan.

### Sample collection

Blood samples (3 mL) were collected from symptomatic patients. Samples testing positive for any malaria parasites by microscopy or for *P. falciparum* (mono-infection or mixed) by a rapid diagnostic test (Fastep Malaria (pf/pv), Carestart ™ Malaria HRP2/pLDH (pf/pv) combo) were included in the study. Fifty microlitres of whole blood was spotted on Whatman 3MM filter paper. Filter papers were dried, sealed in individual plastic bags with a desiccant and kept at room temperature.

### DNA extraction and speciation

Parasite DNA was extracted from filter papers using a previously published extraction method [30]. Extracted DNA was stored at − 80 °C. Speciation was performed by real-time multiplex PCR for *P. falciparum* and *P. vivax* with modifications [31]. PCR-positive samples for *P. falciparum* were used to assess polymorphisms in the genes encoding PfDHFR, PfDHPS and PfK13.

### Pyrosequencing of *pfdhfr* and *pfdhps* mutations

Amplification of a region of the *pfdhfr* gene encoding mutations C50R, N51I, C59R, S108N and I164L and a region of the *pfdhps* gene encoding mutations S436A, A437G, K540E, A581G and A613T/S was conducted by PCR using previously described cycling conditions [32] with a few modifications. Briefly, a 25 µL reaction volume was used for the primary PCR reaction which contained 1 μL of DNA template, 1× PCR buffer (Qiagen, Valencia, CA, USA), 0.2 mM dNTPs (Invitrogen), 1.5 mM magnesium chloride, 0.5 µM of each forward and reverse primer and 0.05 U/µL HotStar Taq DNA polymerase (Qiagen, Valencia, CA, USA). The secondary PCR reaction was prepared in a 50 μL reaction volume, containing 2 µL of primary PCR product, 1× PCR buffer (Qiagen Valencia, CA, USA), 0.2 mM dNTPs (Invitrogen), 1.5 mM magnesium chloride, 0.5 µM of each forward and reverse primer and 0.05 U/µL HotStar Taq DNA polymerase (Qiagen, Valencia, CA, USA). The PCR was performed using a BioRadC1000 or T100 Thermal cycler (Bio-Rad, Hercules, CA). A PyroMark^®^ Q96 MD Pyrosequencer was used for genotyping all codons of interest using a published protocol [32]. 4 μL of secondary PCR product or amplified sequence-specific positive control DNA (BEI Resources, Manassas, Virginia, USA) were used for each pyrosequencing reaction. Single nucleotide polymorphisms (SNPs) were called using PyroMark^®^ Q96 MD pyrosequencing software version 1.2 (Qiagen) in allele quantification mode (AQ) for all SNPs. Peak signal of at least 30 RLU (relative luminescence units) was set as the cut-off for allele quantification. The pyrosequencing reaction was repeated with an adjusted volume of secondary PCR product for samples that failed to produce a SNP call because of either too much or insufficient DNA. Pyrosequencing allele frequencies were adjusted using a standard curve. Standard curve data was generated from pyrosequencing of mixtures of control DNA strains with known proportions of each allele [33]. An allele was considered present in the infection if its frequency after adjustment to the standard curve was above 10%.

### Amplification and sequencing of *pfk13* propeller domain

The gene encoding PfK13 was amplified using Taq2X mastermix (New England BioLabs Inc., USA) in a 25 µL reaction using 12.5 µL of Taq2X mastermix, 1 µL of each 10 µM primer, 5.5 µL nuclease free water and 5 µL template DNA. The PCR thermal cycling conditions for the first round of nested PCR were 94 °C for 5 min followed by 40 cycles of 30 s at 94 °C, 90 s at 54 °C, 90 s at 68 °C and final extension at 68 °C for 10 min. 2 µL of primary PCR product was used as template in the secondary PCR using the same cycling conditions and primer set used previously [26]. PCR products were purified using multiscreen purification plates (Merck Millipore). The sequencing reaction was done using BigDye terminator v3.1 (Thermo Fisher Scientific), with sequencing performed on a 3730xl capillary sequencer (Applied Biosystems). Sequences were edited using Sequencher 5.1^®^ and BioEdit 7.2.6. The sequence from 3D7 strain (PF3D7_1343700) was used as the reference.

## Results

A total of 845 RDT/microscopy-positive samples were tested out of which 300 (238 mono-infection and 62 *P. falciparum* and *P. vivax* mixed infection) samples were PCR-positive for *P. falciparum*. The distribution of *P. falciparum* PCR-positive samples across different regions of Pakistan is listed in Table 1. Participants ranged in age from 1 to 65 years with a median age of 28 years.

**Table 1:** Distribution of samples collected from 16 Pakistani sites

Province	Site	Total samples	PCR-confirmed *P. falciparum*	
			n	**%**
Khyber Pakhtunkhwa (KPK)	Bannu	147	40	27.2
	Hangu	17	14	82.4
	Peshawar	85	14	16.5
	Thall	29	25	86.2
Balochistan	Sui	7	4	57.1
	Quetta	43	29	67.4
	Deramurad Jamali	45	15	31.9
	Zhob	5	4	80.0
FATA^a^	Khyber Agency	74	57	77.0
Punjab	Rawalpindi	16	10	62.5
	Muzafargarh	58	19	32.8
	Multan	106	25	23.6
	Dera Ghazi Khan	23	9	39.1
	Kot Chutta	43	11	25.6
	Rajanpur	45	14	31.1
Sindh	Karachi	102	10	9.8
Total		845	300	35.5

^a^Federally administered tribal areas (FATA) is not a province

### Overall prevalence of PfDHFR and PfDHPS mutations

**Fig. 2:**
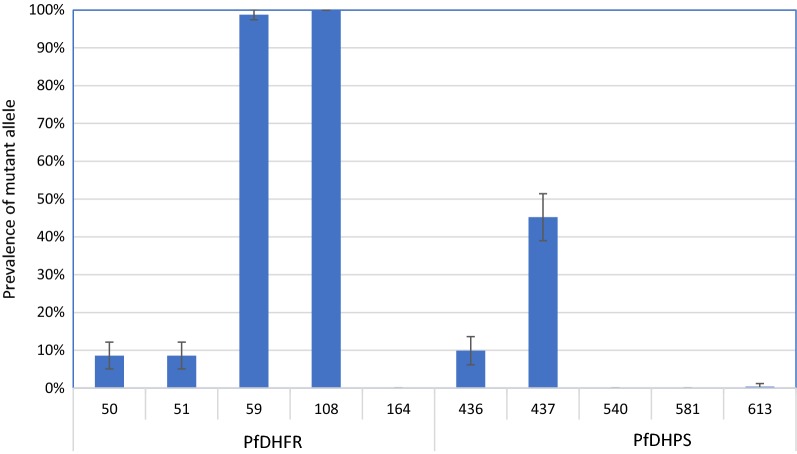
Prevalence of PfDHFR and PfDHPS mutations across all study sites in Pakistan. Amino acid positions are shown on x-axis, while the proportion of infections containing a mutation at a given position is shown on the y-axis. Error bars indicate the standard error

Pyrosequencing of mutations within the genes encoding PfDHFR and PfDHPS revealed that overall, the most prevalent PfDHFR mutation was at amino acid position 108 with 100% of infections containing the 108N allele, followed by the 59R mutation, which was observed in 98.8% of infections. 8.6% of infections contained the 50R and 51I mutations, while no mutations were found at PfDHFR position 164. The most prevalent PfDHPS mutation was 437G, with 45.2% of infections harboring this allele. The prevalence of PfDHPS 436A was 9.9%, and only 0.4% of infections contained parasites with 613T mutation. No mutations were found at PfDHPS positions 540 or 581 (Fig. 2).

### Prevalence of PfDHFR and PfDHPS mutations by region

**Fig. 3:**
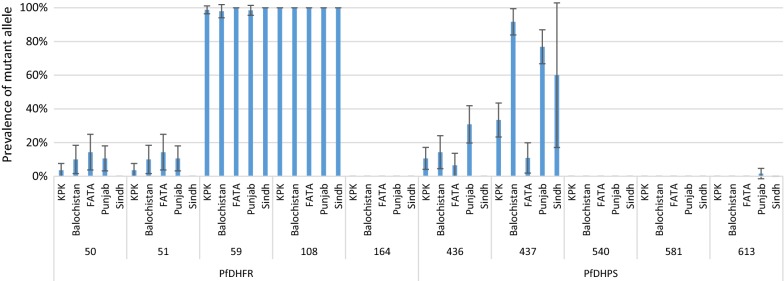
Distribution of PfDHFR and PfDHPS mutant alleles in different regions of Pakistan. Amino acid positions and geographic regions are shown on x-axis, while the proportion of infections containing a mutation at a given position is shown on the y-axis. Error bars indicate the standard error

Across all study sites, 100% of infections contained the PfDHFR 108N mutation. The prevalence of PfDHFR 59R ranged from 98% in Balochistan to 100% in Sindh and FATA. The prevalence of the PfDHFR 50R and 51I mutations ranged from 3.6% to 14.3% in KPK and FATA, respectively, while no mutation at these positions was found in samples from Sindh. PfDHPS 437G had the highest prevalence (91.7%) in Balochistan and the lowest prevalence in FATA (10.9%). The prevalence of PfDHPS 436A ranged from 6.5% to 14.3% in FATA and Balochistan, respectively, while this mutation was not observed in Sindh. The mutation at PfDHPS position 613 was only observed in Punjab with a prevalence of 1.6% (Fig. 3, Additional file 1).

The prevalence of the PfDHFR double mutant (PfDHFR 59R + 108N) was highest (100%) in Sindh, and lowest (93.8%) in Punjab. No PfDHFR triple mutant (51I + 59R + 108N) parasites were found at any site. The prevalence of parasites with PfDHFR 59R + 108N plus PfDHPS 437G was highest (83.3%) in Balochistan and lowest (9.5%) in FATA (Table 2).

**Table 2:** Distribution of mutant PfDHFR and PfDHPS haplotypes of Pakistani *P. falciparum* isolates

	PfDHFR 59R + 108N (n)	PfDHFR 59R + 108N/PfDHPS 437G (n)
KPK	98.8% (83)	28.9% (83)
Balochistan	93.9% (49)	83.3% (48)
FATA	97.6% (42)	9.5% (42)
Punjab	93.8% (64)	45.2% (62)
Sindh	100% (3)	33.3% (3)

### Prevalence of PfK13 mutations

**Fig. 4:**
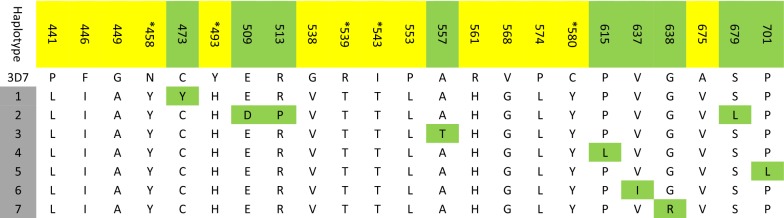
Amino acid sequence alignment of PfK13 haplotypes. Haplotypes observed in this study are highlighted in grey. Numbers in the top row are amino acid positions. Positions highlighted in yellow have been associated with delayed parasite clearance in previous studies, with mutations validated for their role in artemisinin resistance marked with an asterisk. Positions highlighted in green represent non-synonymous mutations identified in this study

Out of 300 PCR-positive samples, 209 were successfully sequenced at *Pfk13*. Nine non-synonymous and four synonymous mutations were observed. The S624S, L678L and S679L PfK13 mutations were unique to Pakistani parasites while the C473Y, E509D, P553P, V637I and G638R PfK13 mutations have been previously reported. The R513P, G544G, A557T, P615L and P701L PfK13 mutations were found to have different amino acid substitutions in samples collected from Pakistan compared to previously reported mutations at these positions from other geographic regions. The prevalence of PfK13 mutations is shown in Table 3. None of the mutations found in this study corresponded to mutations validated to be associated with artemisinin resistance (Fig. 4).

**Table 3:** Prevalence of K13-propeller domain mutations in clinical *P. falciparum* isolates from Pakistan

Mutant codon position	Type of mutation	Prevalence of mutation % (n)	References
C473Y	NS	0.7 (n = 1)	[44]
E509D	NS	0.7 (n = 1)	[47]
*R513P*	NS	0.7 (n = 1)	[48]
*G544G*	S	0.7 (n = 1)	[48]
*P553P*	S	0.96 (n = 2)	[49]
*A557T*	NS	0.7 (n = 1)	[50]
*P615L*	NS	0.7 (n = 1)	[44]
S624S	S	0.7 (n = 1)	*
V637I	NS	0.7 (n = 1)	[51]
G638R	NS	0.7 (n = 1)	[52]
L678L	S	0.7 (n = 1)	*
S679L	NS	0.7 (n = 1)	*
*P701L*	NS	0.7 (n = 1)	[53]

* Mutation not found in literature. Mutations in italics indicate amino acid positions where mutations have been observed previously, but different alleles were present at these positions in the samples from Pakistan compared to those reported in other geographic regions

## Discussion

Anti-malarial drug resistance is a major public health concern and regular monitoring of resistance using molecular markers is essential to track changes in the distribution of resistant parasites and to inform treatment policy. In this study, the prevalence of polymorphisms associated with *P. falciparum* resistance to sulfadoxine–pyrimethamine and artesunate, the drugs included in the first-line ACT in Pakistan, was estimated, namely mutations within the genes encoding PfDHFR, PfDHPS, and PfK13. This study indicates a fixation of the PfDHFR 108N mutation across all study sites, with the PfDHFR double mutant (59R + 108N) near fixation. These results are consistent with those from a study in 2009 that reported a 100% prevalence of this PfDHFR double mutant (59R + 108N) in the Bannu district of the KPK province of Pakistan [19]. Likewise, a study in southern Pakistan reported 87% prevalence of PfDHFR double mutants (59R + 108N) in 2011 [20]. A survey conducted in 2013 across different cities of Pakistan reported an 87% prevalence of the PfDHFR double mutant [21]. Moreover, a therapeutic efficacy study conducted in Zhob, Kech, Khairpur, Thatta, Khrram agency, and Khyber agency also reported near fixation of the PfDHFR double mutant [22]. Similar results have also been reported in the neighboring countries of Iran [34], Afghanistan [35], India [36] and China [37].

The prevalence of the PfDHPS 437G mutation in FATA in this study was 10.9% which is lower than a previously reported prevalence of 38% in samples collected in 2012 from FATA [22]. In addition, the prevalence of parasites with PfDHFR 59R + 108N and PfDHPS 437G was 28.9% in KPK in this study, lower than the previously reported prevalence of 51% in KPK in 2013 [21]. Likewise in FATA the prevalence of this PfDHFR 59R + 108N + PfDHPS 437G mutant was 9.5%, which is lower than the previously reported prevalence of 38% at this site in 2012 [22]. A similar decrease in the prevalence of the PfDHFR 59R + 108N + PfDHPS 437G mutants was observed in Iran where the prevalence of this haplotype dropped from 53.3% in 2008 to 38% in 2010 after the introduction of the ACT AS + SP [38]. Such reductions in the prevalence of SP resistance mutations could possibly be due to reduced SP pressure resulting from the combination of SP with AS and the rapid clearance of *P. falciparum* by the artemisinin component [34].

In contrast, the prevalence of the PfDHPS 437G mutation in this study was higher (91.7%) in Balochistan than reported in previous studies in Balochistan in parasites collected from 2007 to 2012 (70%) [22] and from 2005 to 2007 (60%) [20]. Likewise, the prevalence of the PfDHFR 59R + 108N + PfDHPS 437G mutant in Balochistan was higher in this study (83.3%) than in previous studies that reported prevalences of 69% [22], 58% [21], and 55% in Balochistan [20]. These observations are consistent with findings from Malawi where the prevalence of SP resistance markers remained high 5 years after reducing SP drug pressure following a change in drug policy to use of ACT [33].

The PfDHFR triple mutant (51I + 59R + 108N), quadruple mutant (PfDHFR 51I + 59R + 108N and PfDHPS 437G), and quintuple mutant (PfDHFR 51I + 59R + 108N and PfDHPS 437G + 540E) were not observed in this study. The highly-resistant quintuple mutant has been associated with SP failure [17]. Historically, importation has played a pivotal role in establishing resistance to chloroquine and SP in Africa [39]. Molecular epidemiological studies examining microsatellite markers flanking the *pfdhfr* gene revealed that pyrimethamine resistance was transferred from Southeast Asia to Africa [40]. It has also been shown that PfDHFR mutants in India share ancestry with Southeast Asian PfDHFR mutants [41]. The lack of PfDHFR triple mutants in Pakistan could suggest that this triple mutant parasite has not yet been introduced in Pakistan.

Polymorphisms in the PfK13 propeller domain have been identified as a useful molecular markers for surveillance of artemisinin resistance in Southeast Asia. More than 200 non-synonymous mutations in the PfK13 propeller domain have been reported, but not all these mutations have been associated with artemisinin resistance [42]. N458Y, Y493H, R539T, I543T and C580Y are the molecular markers for artemisinin resistance which have been validated by in vitro and in vivo studies while P441L, F446I, G449A, G538V, P553L, R561H, V568G, P574L, A578S and A675V are candidate markers of artemisinin resistance [42]. This is the first study from Pakistan to estimate the prevalence of PfK13 propeller mutations. Low frequencies of PfK13 propeller mutations (6.2%) were found, and none of the mutations corresponded to SNPs associated with artemisinin resistance in Southeast Asia [26]. A low frequency of PfK13 mutations has been found in countries adjacent to Pakistan [43–45], with the exception of China where a high prevalence of PfK13 mutations has been observed in multiple studies along the China-Myanmar border, F446I being the predominant PfK13 mutation [29, 46]. Absence of PfK13 mutations which are associated with reduced susceptibility to artemisinins suggests that *P. falciparum* is still sensitive to artemisinin-based combination therapy in Pakistan, consistent with the findings of previous therapeutic efficacy studies conducted at sentinel sites in Pakistan from 2007–2012 that demonstrated 98.5–100% adequate clinical and parasitological response [22].

## Conclusion

These findings suggest that the PfDHFR 108N mutation is fixed and PfDHFR double mutation (59R + 108N) is near fixation. In addition, an absence of mutations conferring a high-level of resistance to SP and the artemisinins suggest that artesunate plus SP is still efficacious for the treatment of uncomplicated *P. falciparum* malaria in Pakistan. However, the systematic monitoring of PfDHFR, PfDHPS and PfK13 molecular markers should continue as long as this artemisinin-based combination is used.

## Additional file

**Additional file 1.** Frequency distribution of mutations conferring resistance to sulfadoxine–pyrimethamine in *Plasmodium falciparum* isolates from Pakistan.
