# Supplementary material for: Prevalence of molecular markers of sulfadoxine–pyrimethamine and artemisinin resistance in Plasmodium falciparum from Pakistan
Source: Malar J. 2018 Dec 17;17:471. doi: 10.1186/s12936-018-2620-y (PMC6296135; doi:10.1186/s12936-018-2620-y)
Supplement: Supplementary file 1 — Additional file 1. Frequency distribution of mutations conferring resistance to sulfadoxine–pyrimethamine in Plasmodium falciparum isolates from Pakistan. [file 12936_2018_2620_MOESM1_ESM.docx]

**Additional Table**

**Table S1**. Frequency distribution of mutations conferring resistance to sulfadoxine-pyrimethamine in *Plasmodium falciparum* isolates from Pakistan.

|  |  |  | PfDHFR | | | | |  | PfDHPS | | | | |
| --- | --- | --- | --- | --- | --- | --- | --- | --- | --- | --- | --- | --- | --- |
|  |  |  | 50 | 51 | 59 | 108 | 164 |  | 436 | 437 | 540 | 581 | 613 |
|  |  |  |  |  |  |  |  |  |  |  |  |  |  |
| KPK | Wild |  | 96.4%% | 96.4% | 1.2% | 0% | 100% |  | 89.4% | 66.7% | 100% | 100% | 100% |
|  | Mutant |  | 0% | 0% | 98.8% | 100% | 0% |  | 1.2% | 29.8% | 0% | 0% | 0% |
|  | Mixed |  | 3.6% | 3.6% | 0% | 0% | 0% |  | 9.4% | 3.6% | 0% | 0% | 0% |
|  | n |  | 83 | 83 | 83 | 83 | 83 |  | 85 | 84 | 90 | 84 | 83 |
|  |  |  |  |  |  |  |  |  |  |  |  |  |  |
| Balochistan | Wild |  | 90% | 90% | 2% | 0% | 100% |  | 85.7% | 8.3% | 100% | 100% | 100% |
|  | Mutant MutanMutantaMutant |  | 0% | 0% | 93.9% | 100% | 0% |  | 0% | 89.6% | 0% | 0% | 0% |
|  | Mixed |  | 10% | 10% | 4.1% | 0% | 0% |  | 14.3% | 2.1% | 0% | 0% | 0% |
|  | n |  | 50 | 50 | 49 | 49 | 49 |  | 49 | 48 | 53 | 50 | 49 |
|  |  |  |  |  |  |  |  |  |  |  |  |  |  |
| FATA | Wild |  | 85.7% | 85.7% | 0% | 0% | 100% |  | 93.5% | 89.1% | 100% | 100% | 100% |
|  | Mutant |  | 0% | 0% | 97.7% | 100% | 0% |  | 0% | 10.9% | 0% | 0% | 0% |
|  | Mixed |  | 14.3% | 14.3% | 2.3% | 0% | 0% |  | 6.5% | 0% | 0% | 0% | 0% |
|  | n |  | 42 | 42 | 43 | 43 | 43 |  | 46 | 46 | 53 | 47 | 44 |
|  |  |  |  |  |  |  |  |  |  |  |  |  |  |
| Punjab | Wild |  | 89.4% | 89.4% | 1.5% | 0% | 100% |  | 91% | 50.7% | 100% | 100% | 98.4% |
|  | Mutant |  | 1.5% | 1.5%% | 92.4%% | 100% | 0% |  | 1.5%% | 44.8% | 0% | 0% | 1.6% |
|  | Mixed |  | 9.1% | 9.1% | 6.1% | 0% | 0% |  | 7.5% | 4.5% | 0% | 0% | 0% |
|  | n |  | 66 | 66 | 66 | 64 | 64 |  | 67 | 67 | 70 | 70 | 63 |
|  |  |  |  |  |  |  |  |  |  |  |  |  |  |
| Sindh | Wild |  | 100% | 100% | 0% | 0% | 100% |  | 100% | 40% | 100% | 100% | 100% |
|  | Mutant |  | 0% | 0% | 100% | 100% | 0% |  | 0% | 60% | 0% | 0% | 0% |
|  | Mixed |  | 0% | 0% | 0% | 0% | 0% |  | 0% | 0% | 0% | 0% | 0% |
|  | n |  | 3 | 3 | 3 | 4 | 4 |  | 5 | 5 | 5 | 5 | 4 |
|  |  |  |  |  |  |  |  |  |  |  |  |  |  |
